# Incidence of Leukemia, Lymphoma, and Multiple Myeloma in Czech Uranium Miners: A Case–Cohort Study

**DOI:** 10.1289/ehp.8476

**Published:** 2006-01-26

**Authors:** Vladimír Řeřicha, Michal Kulich, Robert Řeřicha, David L. Shore, Dale P. Sandler

**Affiliations:** 1 Health Institute of the Uranium Industry, Příbram, Czech Republic; 2 Department of Statistics, Faculty of Mathematics and Physics, Charles University, Prague, Czech Republic; 3 Center of Epidemiological Studies, Příbram, Czech Republic; 4 Westat, Inc., Durham, North Carolina, USA; 5 Epidemiology Branch, National Institute of Environmental Health Sciences, National Institutes of Health, Department of Health and Human Services, Research Triangle Park, North Carolina, USA

**Keywords:** chronic lymphocytic leukemia, ionizing radiation, leukemogenesis, occupational exposure, radon

## Abstract

**Objective:**

Uranium miners are chronically exposed to low levels of radon and its progeny. We investigated whether radon exposure is associated with increased incidence of leukemia, lymphoma, or multiple myeloma in this population.

**Design:**

We conducted a retrospective case–cohort study in 23,043 uranium miners and identified a total of 177 incident cases of leukemia, lymphoma, and myeloma. Detailed information on occupational radon exposure was obtained for the cases and a randomly selected subcohort of 2,393 subjects. We used the proportional hazards model with power relative risk (RR) function to estimate and test the effects of cumulative radon exposures on incidence rates.

**Results:**

Incidence of all leukemia combined and chronic lymphocytic leukemia (CLL) alone was positively associated with cumulative radon exposure. The RR comparing high radon exposure [110 working level months (WLM); 80th percentile] to low radon exposure (3 WLM; 20th percentile) was 1.75 [95% confidence interval (CI), 1.10–2.78; *p* = 0.014] for all leukemia combined and 1.98 (95% CI, 1.10–3.59; *p* = 0.016) for CLL. Myeloid leukemia and Hodgkin lymphoma were also associated with radon, but RRs were not statistically significant. There was no apparent association of radon with either non-Hodgkin lymphoma or multiple myeloma. Exposure to radon and its progeny was associated with an increased risk of developing leukemia in underground uranium miners. CLL, not previously believed to be radiogenic, was linked to radon exposure.

Ionizing radiation is a known carcinogen. Uranium miners are at risk of developing radiation-related cancer because they are chronically exposed to alpha particles emitted by radon and its progeny (henceforth referred to as radon) and to low levels of whole-body gamma radiation. Both types of radiation can cause cellular damage and, in some conditions, induce malignant tumors.

Based on epidemiologic studies in uranium miners, it has long been recognized that radon causes lung cancer [National Research Council (NRC) 1988, 1999; Řeřicha and Šnajberk 1966]. It is unclear whether radon causes other cancers, including those of the hematopoietic system. Several ecologic studies (Laurier et al. 2001) reported an association between indoor radon exposures and leukemia. Myeloid leukemia and acute lymphocytic leukemia (ALL) were linked to external radiation in Japanese atomic bomb survivor studies (Pierce and Preston 2000; Preston et al. 1994). A similar association was demonstrated in patients exposed to therapeutic and diagnostic irradiation [Hart and Wall 2002; NRC 1990; Ron 2003; United Nations Scientific Committee on the Effects of Atomic Radiation (UNSCEAR) 2000]. Studies in nuclear worker cohorts show some evidence of an association with leukemia excluding chronic lymphocytic leukemia (CLL) (Cardis et al. 2005; Muirhead et al. 1999; Sont et al. 2001; UNSCEAR 2000). An association of CLL with radiation exposure has not been demonstrated.

Several issues limit the conclusions that can be drawn from past epidemiologic studies. The ecologic studies are methodologically limited, and their results are inconsistent and unconvincing (Laurier et al. 2001). More rigorous case–control studies (Law et al. 2000; Lubin et al. 1998) and cohort studies in uranium miners (Darby et al. 1995) have not consistently demonstrated increased risks for hematopoietic malignancies. Studies in atomic bomb survivors apply to instantaneous uniform whole-body external radiation with a wide range of doses. Therapeutic partial-body irradiation uses mainly high doses of external radiation. Diagnostic irradiation usually uses low external doses; in the case of Thorotrast (Andersson et al. 1993), a contrast material, the radiation is internally transmitted by alpha emitters. Nuclear workers are exposed to protracted low levels of whole-body external radiation, sometimes combined with internal exposure to long-lived radionuclides and/or chemicals.

Most studies, including all studies of miners, investigated mortality rather than cancer incidence. Mortality is a good surrogate for cancer incidence when investigating cancers with poor survival, such as lung cancer. However, a substantial number of cases are likely to be missed in mortality studies of cancers with low fatality rates, such as CLL. Some studies (e.g., Dupree-Ellis et al. 2000) reported only standardized mortality ratios comparing an occupational cohort with a general population. Such analyses do not capture the dose–response relationship well and are prone to bias because of differences between the cohort and the population at large. Other studies lacked sufficient power because of small numbers of cases or short follow-up periods (Dupree-Ellis et al. 2000; Tirmarche et al. 1993). A common problem is inaccurate or incomplete radiation exposure measurements (Tomášek and Darby 1995).

The Health Institute of the Uranium Industry (HIUI) and the National Institute of Environmental Health Sciences (NIEHS) initiated a study of cancer incidence in a cohort of Czech uranium miners exposed to radon (Řeřicha et al. 1998). A significant increase in leukemia risk was observed among miners employed for at least 1 year underground compared with the incidence in the general Czech population, with a standardized incidence ratio of 1.58 [95% confidence interval (CI), 1.17–2.16], based on 41 cases. Risk appeared to increase with increasing radon exposure. This case–cohort analysis was undertaken to extend the follow-up period (adding additional cases), to refine the diagnostic information, and to improve the estimation of exposures.

## Methods

Uranium mines in the Příbram region of the Czech Republic, about 60 km southwest of Prague, were the largest in the former Czechoslovakia. The employment registry of the Příbram Uranium Industry (UI) lists nearly 48,000 employees who ever worked in the Příbram mines during the operating period (1949–1991). Our cohort consisted of male workers who 1) were listed in the UI employment registry at any time between 1 January 1949 and 31 December 1975; 2) had registry records indicating that they had worked underground for at least 1 month before 1 January 1976; and 3) lived in the former Czechoslovakia on 1 January 1977. All 27,441 workers who satisfied criteria 1 and 2 were matched against the National Registry of Inhabitants and the National Pension Funds registry to check their vital status as of 1 January 1977. The matching success rate was 92%. The study cohort included 23,043 male workers who satisfied criteria 1–3. The study protocol was reviewed by the chair of the Institutional Review Board (IRB) at the NIEHS and determined to be exempt from full IRB review because it involved linkage of existing records and analysis of coded data without identifiers.

### Cases and subcohort

This study used a stratified case–cohort sampling design. All incident solid cancers, lymphomas, and myelomas occurring in the cohort between 1 January 1977 and 31 December 1996, as well as all incident leukemias occurring between 1 January 1977 and 31 December 2001, were identified by matching the 23,043 cohort subjects with the Czech and Slovak national cancer registries. Reporting of cancer cases to the registries was mandatory. Study subjects were matched to the incidence records using unique government-assigned identification numbers. A total of 2,949 incident cancers of all types were identified in the cohort; among them were 177 cases of leukemia, lymphoma, or multiple myeloma. All cancers were recoded according to the *International Classification of Diseases, Ninth Revision* (ICD-9; World Health Organization 1979). We selected a subcohort of 2,393 subjects by stratified random sampling. Stratification factors were age on 1 January 1977 (5-year groups) and employment duration (< 1 year vs. ≥ 1 year). Subcohort sampling fractions were proportional to the number of cancer cases identified in the stratum, resulting in a subcohort that was, by design, older than the cohort as a whole (because cancer cases tended to be older).

### Employment history

The HIUI and the Uranium Industry Concern provided employment logs for the cases and the subcohort subjects. These records contained detailed work history, including information on exact duration of employment in different professions and workplaces, which was used to estimate radiation exposures. Miners could have worked in one or more professional capacities, with varying exposures to radiation.

### Radon exposure

Individual exposures to radon for the period 1949–1967 were assigned using industry tables of mean annual ^222^Rn concentrations in picocuries per liter for each mine. The tables were based on a total of > 50,000 radon gas measurements systematically covering locations scattered throughout the mines. An equilibrium factor was applied to convert radon gas concentration in picocuries per liter to the potential alpha energy of radon progeny. The equilibrium factor values usually ranged from 0.4 to 0.6; they were calculated from a series of measurements taken in the mines, reflecting changes in ventilation conditions. The exposure to radon progeny was calculated from the potential energy measurement taken in the workplace during the period of interest, the duration of the period, and the proportion of working time spent underground. Since 1968, over 190,000 direct measurements of the potential alpha energy of radon progeny were obtained. Radon exposures were converted to working level months (WLM). A working level is the concentration of any combination of the short-lived radon progeny in 1 L of air that will result in the estimated emission of 1.3 × 105 MeV of potential alpha energy. A WLM is defined as exposure to 1 WL of radon for 170 hr.

### Other occupational exposures

Since 1966, individual film badge measurements of gamma exposure were recorded for a majority of underground workers. We developed a statistical model to estimate missing gamma exposures in the period before 1966 from annual ore productivity level, calendar year, number of shifts worked, and profession. Long-lived radionuclides formed a negligible part of the total aerial activity; their concentration in the mine air was < 0.1 Bq/m^3^. Dust measurements were recorded monthly for each workplace. Mean concentrations of airborne dust reached 10 mg/m^3^ in the 1950s, dropped to 2–4 mg/m^3^ in the 1960s, and later stabilized to < 2 mg/m^3^. The concentration of arsenic in Příbram ore was low; the average content of arsenic in samples of load ore was 25 mg/kg, about 200 times less than in Joachimsthal, another West Bohemian mining region. The workers were not exposed to diesel fumes; all underground transportation used electric power.

### Smoking status

The sources of smoking information were records from job-entry medical examinations taken from HIUI archives and records from mandatory annual preventive medical check-ups. The subjects were classified as *a*) never smokers and light smokers who smoked < 10 cigarettes a day for up to 5 years; and *b*) moderate and heavy smokers who exceeded the limit for light smokers.

### Cancer diagnosis

Hematopoietic cancer diagnoses reported to the registry were made by regional hematology specialists using methods valid at the time of the diagnosis. After 1990, criteria recommended by the French–American–British cooperative group, the International Workshop on CLL, and the National Cancer Institute Working Group were used. When hematologists in regional hospitals could not verify the original diagnosis, patients were referred to university hematology departments for immunophenotyping.

### Statistical methods

Miners were followed from 1 January 1977 until the first recorded occurrence of a cancer of interest, death, or end of follow-up, whichever occurred first. The end of the follow-up was 31 December 2001 for analyses involving only leukemia and 31 December 1996 for all other analyses. Radon exposures were cumulated over the working histories with a 2-year lag.

In analyses involving risk modeling, cases received a weight of 1 and subcohort controls the inverse empirical sampling fraction applicable to their stratum (Borgan et al. 2000). In categorical analyses, age was categorized into 5-year groups. The number of cases expected under no exposure effect was calculated from weighted person-years at age-exposure cells and observed incidence rates in age groups. The age-standardized incidence rate was calculated from observed incidence rates in age-exposure cells and weighted person-years accrued in age groups as proportions of the total person-years for the whole cohort.

The hypothesis of no association between radiation exposure and cancer incidence was tested using a proportional hazards model with age as the time scale and the power relative risk function RR(*y*) = (1 + *y*)^β^, where *y* is lagged cumulative exposure, RR(*y*) is RR of exposure *y* compared with zero, and β is a parameter to be estimated. We report the estimated effects in the form of RRs comparing the 80th and 20th percentiles of the distribution of cumulative lifetime exposure in the study population; that is, RR(*y_80_,y_20_*) = [(1 + *y_80_*)/(1 + *y_20_*)]^β^. All analyses were adjusted for smoking. Exposure effects were tested using a pseudoscore test. CIs were based on estimated parameters and their standard errors. The effect of time since exposure was incorporated by dividing the total exposure into three exposure variables (exposure acquired 2–15 years ago, 15–25 years ago, and > 25 years ago).

Parameters in the linear excess RR (ERR) model were estimated by maximizing Poisson-like pseudolikelihood for a table classifying cases and weighted person-years by age and exposure groups. Separate age effects were estimated for each age group. This method yields valid estimates of the linear ERR parameter but no valid tests or CIs. There is currently no software to get valid tests and standard errors for the linear ERR parameter from a stratified case–cohort study.

The analyses were done in R (R Foundation for Statistical Computing, Vienna, Austria) and Matlab (MathWorks, Natick, MA, USA). Proportional hazards models were fitted using a Fortran program interfaced to MATLAB. Primary analyses were verified in SAS (SAS Institute, Inc., Cary, NC, USA).

## Results

Complete exposure and follow-up histories were determined for 177 cases and 2,393 subcohort subjects. The total study population was 2,558 because of a small overlap between the cases and the subcohort. Table 1 shows the cohort, the subcohort, and the cases classified according to the sampling strata. Most workers in the cohort were ≤ 45 years of age in 1977. Because of stratified sampling with respect to age, the subcohort included relatively older subjects. The mean age ± SD of the study subjects at the start of follow-up was 50 ± 10.6 years. The median follow-up duration was 18.8 years.

Among hematopoietic malignancies, the most frequent diagnosis (Table 2) was CLL, followed by non-Hodgkin lymphoma, multiple myeloma, Hodgkin lymphoma, and other diagnoses. The average age at diagnosis was 60.1 years (range, 28.4–86.4 years).

The mean ± SD duration of employment was 6 ± 6.6 years (range, 1 month–39 years). Over 46% of the workers began employment before 1955; < 10% were still employed at the start of follow-up. The mean ± SD lifetime radon exposure was 64.1 ± 98 WLM (median, 23.2 WLM; range, 0–959 WLM). Annual radon exposure rates decreased over time because of improved ventilation and other protective measures. The mean annual exposure rate was 29.3 WLM/year in 1949–1955, 11.7 WLM/year in 1956–1965, 2.9 WLM/year in 1966–1975, and 0.7 WLM/year in 1976–1992. Consequently, younger subjects had much lower radon exposures than older subjects (Table 1). Over 69% of the subjects were classified as moderate and heavy smokers.

Table 3 shows observed numbers of cases of each type of cancer by radon exposure category, along with the ratio of observed numbers and expected numbers under the assumption of no association of incidence with exposure. Table 3 also presents incidence rates standardized to the age distribution of the whole cohort. For all hematopoietic cancers combined, there was no consistent pattern in incidence rates with increasing exposure. For all leukemia types combined and for CLL, the rates were not entirely consistent but generally rose with increasing WLM.

In proportional hazards analysis, the risk of all leukemias combined and the risk of CLL specifically was significantly associated with radon exposure (Table 4). To evaluate the associations, we transformed the power risk parameters to reflect the risks for the upper exposure quintile (110 WLM) relative to the lower quintile (3 WLM). These estimated risks were 1.75 for leukemia and 1.98 for CLL, with CIs excluding 1. The RR for Hodgkin lymphoma and myeloid leukemia was also elevated but was not statistically significant. Non-Hodgkin lymphoma and multiple myeloma were not associated with radon. The analysis of all lymphocytic leukemias combined is not presented because this group included only 3 non-CLL cases. Separate analyses of ALL and acute and chronic myeloid leukemia were not performed because the number of cases was too small.

**Figure 1:**
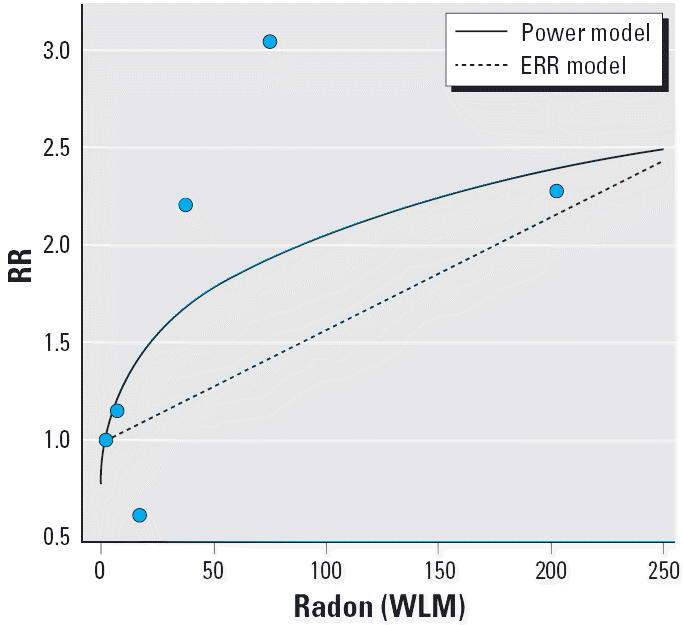
Comparison of power RR and linear ERR fits based on the grouped Poisson model. The points mark empirical RRs for the exposure groups; the curves are normalized to compare exposures with the midpoint of the lowest exposure group (2.5 WLM).

We used the grouped Poisson model to estimate linear ERR parameters to compare the power risk model to the linear ERR model. RRs from the linear ERR model were similar but slightly attenuated. For example, the estimated RR comparing 110 WLM to 3 WLM was 1.46 for all leukemia combined and 1.63 for CLL (results not shown). Figure 1 shows that the power model gives higher radon risk estimates for CLL than the linear ERR model but has smaller residuals at the higher exposure groups. Maximized Poisson pseudolikelihoods for both models were very close to each other, indicating that the goodness of fit was about the same.

Possible confounding and effect modification by other factors was also investigated. Calendar year of follow-up, age at the start of exposure, exposure rates, and year of start of employment did not affect the incidence rates or modify the effects of radon exposure. However, we found that the association of radon with leukemia and CLL significantly decreased with time since exposure. For example, the estimated RR for CLL comparing 110 WLM with 3 WLM was 1.15 (95% CI, 0.68–1.94; *p* = 0.61) for exposures acquired > 25 years ago, 1.93 (95% CI, 1.17–3.19; *p* = 0.024) for exposures acquired 15–25 years ago, and 4.20 (95% CI, 1.84–9.61; *p* = 0.02) for most recent exposures (2–15 years ago).

## Discussion

We found a significant relationship between cumulative radon exposure and incidence of leukemia, especially CLL. For myeloid leukemia and Hodgkin disease, the risk was elevated but nonsignificant. We saw no association of radon with the incidence of non-Hodgkin lymphoma or multiple myeloma.

Miners are also exposed to gamma radiation, which is associated with non-CLL leukemia. In the mines, gamma radiation and radon exposure are correlated, and the observed risk associated with radon could be explained by exposure to gamma irradiation. In our study, the estimated lifetime gamma exposures were relatively low (mean ± SD, 10.3 ± 14.3 mGy; range, 0–227 mGy) and highly correlated with cumulative radon exposures (correlation 0.80 on the log scale). We did a parallel analysis of gamma exposure and found a significant association with all leukemias combined (RR = 1.63; 95% CI, 1.05–2.54; *p* = 0.03) and CLL (RR = 1.96; 95% CI, 1.12–3.42; *p* = 0.02) when comparing exposures in the 80th percentile (20 mGy) to the 20th percentile (1 mGy). This result seems implausible at this low dose, although we cannot rule out such an effect, perhaps in combination with an effect due to radon. There are two potential alternative explanations: either the elevated risk with gamma reflects the strong correlation with radon, or our estimates of pre-1966 gamma exposures were too low. We have no data to verify our ore-productivity–based estimates of gamma exposures before 1966. It is unlikely that non-radiation exposures were responsible for the observed risk, because the workers in Příbram were not exposed to any other substances suspected of causing leukemia.

Smoking was not associated with any hematopoietic malignancy and did not appear to confound the association between radon and leukemia. The estimated RRs due to smoking ranged from 0.83 to 1.72 with wide CIs. Smoking effects could not be evaluated with much precision because smoking was very common and our data on smoking were limited.

The primary strengths of this study are *a*) the use of incidence rather than mortality as the outcome; *b*) the completeness and quality of the national cancer registries; *c*) the availability of detailed work histories; *d*) radon exposure data based on almost 250,000 radon measurements in the workplaces; and *e*) statistical methods that do not lose information by grouping age and exposures. The case–cohort design allowed an exceptionally detailed evaluation of exposures based on employment logs of the cases and subcohort members. Beginning follow-up after the end of the exposure period for most miners made this study particularly suitable for detecting long-term effects of exposure.

This study has several limitations, although none seem to account for the observed association between radon exposure and leukemia. We had no information on cancer diagnoses before 1977 and may have missed some cases of ALL, in particular. Furthermore, someone with leukemia diagnosed before 1977 could have entered the study as a subcohort control. The incidence of CLL would be affected more than that of other cancers because of the long survival of CLL patients. Such subjects would be older, on average, than our study cohort, and their radon exposures would be higher. Thus, including many unrecognized pre-1977 CLL cases would bias the observed effect of radon on CLL downward. In addition, diagnosis of CLL can be delayed because the disease often remains asymptomatic for a long time. However, all UI workers underwent annual medical examinations (including blood analyses) during their employment and were also examined, although less frequently, in retirement. Thus, it is unlikely that a substantial number of unrecognized CLL cases were included in the subcohort.

The exposure measurements are subject to measurement error, which is largest for high exposures acquired in the early period. The analyses are not adjusted for exposure measurement errors. These errors typically attenuate an existing exposure effect rather than induce an effect where there is none. It is reassuring, however, that the exposure effects were strongest in the most recent period. The conclusions hold even if the high exposures acquired in the early mining period are eliminated.

Many studies of radiation–cancer associations fit the ERR model with a linear dose–response relationship. We chose the power RR model because the linear ERR model tends to suffer from unreliable standard errors and poor power (Breslow and Day 1987). The power RR model does not share the problems of the ERR model, can be used with ungrouped data, and provides better power and more reliable *p*-values and CIs. The goodness of fit was similar for both models.

In the past, radon was not considered a potential cause of leukemia because it was believed that most of its alpha activity was released in the respiratory tract. However, several authors (e.g., Henshaw et al. 1990; Richardson et al. 1991) have suggested that radon inhalation may deliver a non-negligible alpha radiation dose to bone marrow and thus increase leukemia risk. Inhaled radionuclides may transmit their alpha activity directly after reaching bone marrow via the blood stream. Because of its high solubility in fat, radon gas itself may concentrate in bone marrow fat cells and emit alpha particles to the surrounding hematopoietic stem cells (Allen et al. 1995). Recently published models that simulated radiation dose to red bone marrow after inhalation of radon attributed a much more important role to radon gas than to radon progeny (Kendall and Smith 2002). Thus, it is plausible that radon causes much of the observed increased risk of leukemia.

Our findings challenge the idea that CLL is not radiation related. Although well accepted, the evidence supporting this notion is not persuasive (Richardson et al. 2005). CLL etiology is still unknown, but the pathogenesis of CLL appears to be similar to other leukemia subtypes. Genetic alterations, inherited or acquired, are likely to lead to any type of leukemia. Like other subtypes, CLL is associated with specific chromosome and immune system alterations. Altered apoptosis is likely to be involved in CLL leukemogenesis. Mutations of tumor suppressor genes, especially *p53*, and an upregulated expression of the *bcl-2* gene may have an important role in the inhibition of apoptosis (Irons and Stillman 1996; Stilgenbauer et al. 2002). Ionizing radiation produces biologic damage that may play a role in one or more stages of the neoplastic process leading to CLL. There is no evidence from research at the molecular level that CLL cannot be induced by ionizing radiation.

CLL is the most frequent type of leukemia among adults in Western Europe and North America. It is extremely rare in East Asia. Therefore, studies of the Japanese atomic bombing cohorts had limited power to detect an association with CLL. CLL may remain asymptomatic for years. Patients often have a prolonged, nonaggressive course marked only by a stabilized lymphocytosis in the peripheral blood and bone marrow. It is not unusual for CLL to be revealed by chance during a consultation for apparently unrelated disease or during a routine examination. Many patients with CLL require no specific treatment and die from other diseases typical for old age (Greer et al. 2003; Henderson et al. 2002).

Given the characteristics of CLL, a substantial number of cases will be missed in studies that rely on death certificates. The accuracy of death certificates compared with hospital and autopsy diagnoses in the same cohort has been shown repeatedly to be poor, especially for diagnoses with low case-fatality. For example, Demers et al. (1992) compared death certificates and autopsy data and found that only 2 of 24 autopsy-diagnosed cases were reported on the death certificates. In another study, Percy et al. (1990) compared underlying causes of death based on death certificates with hospital diagnoses; only 65% of death certificate-based diagnoses were consistent with hospital records. In a study of deaths among atomic bomb survivors, the overall agreement between death certificates and autopsy diagnoses was only 52% (Ron et al. 1994). Thus, mortality studies investigating diseases with low fatality rates, such as CLL, are likely to miss exposure effects because of poor ascertainment of cases and competing causes of death. All previous studies of uranium miner cohorts collected only mortality data based on death certificates.

To overcome the limitations of mortality studies, we studied incidence of hematopoietic malignancies in uranium miners and found a significant association between cumulative radon exposure and incidence of all leukemias, especially CLL. Our findings support the hypothesis that CLL is also radiogenic (Richardson et al. 2005). If this conclusion is confirmed by future studies, leukemia, including CLL, should be considered an occupational disease in workers with prolonged low-level exposure to radon and perhaps to other kinds of radiation.

## Figures and Tables

**Table 1:** Cohort and subcohort subjects classified by sampling strata: duration of employment and age and lifetime radon exposure by age.

	No. (%) of subjects by duration of employment						
	≥ 12 months			< 12 months			
Age group^a^ (years)	Cohort	Subcohort	Cases	Cohort	Subcohort	Cases	Mean radon^b^ (WLM)
19–35	6,857 (41.0)	130 (7.1)	23 (17.6)	2,848 (45.0)	103 (18.2)	9 (19.6)	5.9
36–45	3,336 (20.0)	338 (18.5)	19 (14.5)	1,484 (23.5)	135 (23.8)	15 (32.6)	37.4
46–55	4,057 (24.3)	792 (43.4)	57 (43.5)	1,385 (21.9)	234 (41.3)	18 (39.1)	78.4
56–65	1,756 (10.5)	447 (24.5)	28 (21.4)	449 (7.1)	74 (13.1)	1 (2.2)	83.0
66–90	715 (4.3)	119 (6.5)	4 (3.1)	156 (2.5)	21 (3.7)	3 (6.5)	83.7
Total	16,721 (100)	1,826 (100)	131 (100)	6,322 (100)	567 (100)	46 (100)	64.1

a Age on 1 January 1977.

b Mean lifetime exposure for the cases and subcohort subjects.

**Table 2:** Incidence of diagnoses.

		Follow-up period (no. of cases)		
Diagnosis	ICD-9 code^a^	1977–1996	1997–2001	Total
Lymphosarcoma, reticulosarcoma	200	11	—b	11
Hodgkin lymphoma	201	23	—b	23
Non-Hodgkin lymphoma	202	33	—b	33
Multiple myeloma	203	26	—b	26
ALL	204.0	2	0	2
CLL	204.1	40	13	53
Aleukemic leukemia	204.8	1	0	1
Acute myeloid leukemia	205.0	14	2	16
Chronic myeloid leukemia	205.1	7	1	8
Other myeloid leukemia	205.8	0	1	1
Other leukemia	207	2	0	2
Unspecified leukemia	208	1	0	1
Total	—	160	17	177

a World Health Organization (1979).

b Follow-up was terminated in 1996.

**Table 3:** Numbers of cases and age-standardized incidence rates by radon exposure category.

	Radon (WLM)						
Diagnosis (ICD-9 code)	0–5	5–10	10–25	25–50	50–100	> 100	Total
All (200–208)^a^							
Cases	41	22	21	13	24	39	160
O/E^b^	0.90	1.19	0.83	0.77	1.15	1.18	
Rate^c^	32.8	47.8	33.5	27.7	39.9	43.3	39.5
Hodgkin lymphoma (201)^a^							
Cases	9	1	4	2	3	4	23
O/E^b^	0.92	0.35	1.14	1.03	1.43	1.43	
Rate^c^	3.3	1.8	5.4	3.7	5.7	3.6	5.0
Non-Hodgkin lymphoma (200, 202)^a^,^d^							
Cases	18	9	4	6	3	4	44
O/E^b^	1.10	1.66	0.57	1.44	0.65	0.62	
Rate^c^	11.7	16.0	6.0	10.9	4.6	7.1	9.4
Myeloma (203)^a^							
Cases	4	5	4	1	5	7	26
O/E^b^	0.69	1.70	0.98	0.33	1.29	1.11	
Rate^c^	4.7	11.7	6.7	2.1	8.1	6.7	6.7
Leukemia (204–208)							
Cases	17	8	12	7	16	24	84
O/E^b^	0.76	0.85	0.90	0.75	1.39	1.35	
Rate^c^	13.1	15.3	15.6	13.6	24.2	26.7	17.8
CLL (204.1)							
Cases	11	2	6	6	12	16	53
O/E^b^	0.82	0.34	0.72	0.99	1.60	1.37	
Rate^c^	9.4	3.9	7.9	10.5	18.8	17.4	11.4
Myeloid leukemia (205)							
Cases	5	3	5	1	4	7	25
O/E^b^	0.66	1.06	1.22	0.38	1.27	1.47	
Rate^c^	3.1	5.8	6.6	3.1	5.7	9.0	5.4

a Follow-up ended on 13 December 1996.

b Observed number of cases divided by that expected under no exposure effect.

c Age-standardized incidence rate per 100,000 person-years.

d Including lymphosarcoma/reticulosarcoma.

**Table 4:** Associations of radon exposure to incidence of lymphoma, myeloma, and leukemia.

Outcome	Cases (*n*)	RR	(95%CI)^a^	*p*^b^
All	160	1.22	(0.87–1.72)	0.24
Hodgkin lymphoma	23	2.12	(0.81–5.52)	0.12
Non-Hodgkin lymphoma^c^	44	0.80	(0.46–1.37)	0.4
Myeloma	26	1.03	(0.47–2.27)	0.94
Leukemia	84	1.75	(1.10–2.78)	0.014
CLL	53	1.98	(1.10–3.59)	0.016
Myeloid leukemia	25	1.86	(0.79–4.36)	0.14

The reported RRs compare 110 WLM (80th percentile of the cumulative lifetime dose) to 3 WLM (20th percentile).

a 95% CI based on estimated parameter and SE.

b *p*-Value for the hypothesis of no exposure effect (RR = 1) based on pseudoscore test in the power risk model.

c Including lymphosarcoma/reticulosarcoma.
